# Integrating Gene Expression With Recurrent Mutations Improves Age‐Stratified Risk Prediction in Acute Myeloid Leukemia

**DOI:** 10.1002/jha2.70261

**Published:** 2026-03-11

**Authors:** Mobina Shrestha, Salina Dahal, Asis Shrestha, Patricia McNally, Amir Babu Shrestha, Niklas Mackler

**Affiliations:** ^1^ USACS Sentara Albemarle Medical Center Elizabeth City North Carolina USA; ^2^ The Ohio State University Columbus Ohio USA; ^3^ University of Arkansas for Medical Sciences Little Rock Arkansas USA; ^4^ Trinity Health Ann Arbor Ypsilanti Michigan USA; ^5^ Manipal College of Medical Sciences Pokhara Nepal

## Abstract

**Background:**

Older adults with acute myeloid leukemia (AML) have inferior outcomes, yet current genetic risk models do not explicitly account for how age modifies the prognostic impact of molecular features. We hypothesized that integrating apoptosis and p53‐related gene expression with recurrent mutations would improve prediction of complete remission (CR) and 2‐year overall survival (OS), particularly across age groups.

**Methods:**

Using the BeatAML2 dataset (805 patients; 942 specimens), we built two cohorts: a clinical cohort of 916 adults with full data and an expression‐linked cohort of 852 with matched RNA‐seq. Patients were divided into four age groups 18–30, 30–45, 45–60, and 60+ years. We tested whether adding expression of 12 apoptosis and p53‐related genes to five well‐known mutations, that is TP53, NPM1, FLT3, RUNX1, and ASXL1, could improve the prediction of CR and 2‐year OS.

**Results:**

Adding gene expression improved predictive performance across models. For 2‐year OS, AUCs rose from 0.765 to 0.772 in XGBoost, 0.703 to 0.843 in Random Forest, and 0.697 to 0.721 in Logistic Regression. For CR, performance improved from 0.770 to 0.851 in XGBoost, 0.811 to 0.861 in Random Forest, and 0.731 to 0.696 in Logistic Regression. Calibration was strongest for tree‐based models, and reclassification improved most with XGBoost. Multivariable regression confirmed TP53 as the most adverse marker for OS (HR: 3.07), with added risk from ASXL1 (HR: 1.53) and FLT3 (HR: 1.39). NPM1 increased the chance of remission (OR: 2.47) but did not extend survival. SHAP analysis showed that age remained the leading predictor of OS. Among genes, CHEK2 expression was most important for survival, especially in patients 60 years and older, while CCNG1 expression best predicted remission, along with BAX and MCL1.

**Conclusions:**

These results demonstrate that combining gene expression with recurrent mutations makes risk prediction more accurate, especially in older patients who formed the largest group and had the poorest outcomes. Although treatment variables were not included and analysis focused on selected genes, these findings support incorporation of expression‐based features into genetic risk models and warrant prospective validation.

**Trial Registration:**

The authors have confirmed clinical trial registration is not needed for this submission

## 1 Introduction

Acute myeloid leukemia (AML) is an aggressive hematologic malignancy characterized by substantial genetic and clinical heterogeneity. Patient age is among the strongest prognostic factors, with older adults experiencing higher treatment‐related mortality, lower remission rates, and shorter overall survival (OS) than younger patients [1, 2]. This disparity reflects both the accumulation of adverse‐risk mutations and the increased prevalence of chemo‐resistant disease biology in older age groups [3, 4]. Modern prognostic systems incorporate cytogenetic and mutational data to guide risk stratification and treatment selection [5, 6]. However, these models typically adjust for age as a covariate rather than accounting for its potential to modify the prognostic weight of molecular features. Understanding whether specific genetic or transcriptomic markers have different implications across age groups could improve personalized risk assessment in AML.

Apoptosis dysregulation plays a central role in AML pathogenesis and treatment resistance. Members of the BCL2 family tightly regulate the intrinsic apoptotic pathway, balancing pro‐survival proteins such as BCL2, MCL1, and BCL2L1 against pro‐apoptotic effectors including BAX, BCL2L11, and PMAIP1 [7, 8]. Overexpression of anti‐apoptotic proteins can confer resistance to conventional chemotherapy and targeted agents [9]. Venetoclax, a selective BCL2 inhibitor, has significantly improved outcomes in older or unfit AML patients when combined with azacitidine or low‐dose cytarabine [10, 11], yet primary and acquired resistance remain frequent, often mediated by MCL1 or BCL2L1 upregulation [12, 13]. Preclinical studies suggest that gene expression‐based apoptotic signatures may predict therapy response and survival [14, 15], but these observations have rarely been validated in large, clinically annotated AML cohorts.

The BeatAML2 initiative provides a comprehensive resource for tackling these gaps, with integrated genomic, transcriptomic, and clinical outcome data from 805 AML patients and 942 specimens [16]. Therefore, in this study, we used the BeatAML2 dataset to test whether adding expression of apoptosis and p53‐pathway genes to established mutations (TP53, NPM1, RUNX1, ASXL1, and FLT3) and age groups could improve prediction of complete remission (CR) and 2‐year OS. We applied machine learning models with SHAP‐based interpretation to uncover age‐specific patterns of prognostic importance and validated these findings using multivariable Cox regression. Our results show that considering both genetic and gene expression features in an age‐specific framework reveals distinct prognostic patterns, with important implications for personalized AML risk assessment and treatment decisions.

## 2 Methods

### 2.1 BeatAML2 Patient Cohort and Clinical Endpoints

We conducted a retrospective analysis of adults diagnosed with AML using the BeatAML2 dataset, which was prospectively collected at Oregon Health & Science University and integrates clinical outcomes with genomic, transcriptomic, and ex vivo drug sensitivity testing [17]. Out of the 942 available specimens from 805 patients, we selected 916 specimens with complete data on age, mutation status, induction response, OS, and vital status to form the clinical cohort. Of these, 852 specimens also matched RNA‐sequencing (RNA‐seq) profiles, constituting the expression‐linked cohort. The age at diagnosis was grouped into four groups that is 18–30, 30–45, 45–60, and 60+ years. Since the BeatAML2 dataset is specimen‐based, some patients contributed more than one molecular profiling specimen obtained at different clinical timepoints, therefore, our analysis was performed at the specimen level while maintaining patient linkage for survival outcomes. The dataset's integration of genetic, transcriptional, and clinical data within the same patient population made it particularly well‐suited to evaluating the relationship between molecular features, age, and treatment outcomes. We defined OS as the time from diagnosis to death from any cause, with patients censored at their last known follow‐up if alive. For secondary analysis, we also evaluated 2‐year OS, indicating whether patients survived at least 2 years after diagnosis. We selected 2‐year OS as the primary survival endpoint because most AML‐related mortality occurs within the first 2 years after diagnosis, making this interval a clinically meaningful timeframe for risk prediction. This endpoint has also been widely used in AML prognostic modeling studies to evaluate early treatment response and relapse risk. To ensure that findings were not dependent on categorical age grouping, additional sensitivity analyses were performed, treating age as a continuous variable in multivariable survival models. Treatment regimens reflected real‐world clinical practice and included both intensive induction chemotherapy and lower‐intensity approaches, including venetoclax‐based regimens. Treatment exposure was not incorporated into predictive modeling because the primary objective of this study was to evaluate baseline molecular determinants of remission and survival. CR was recorded when there was documented morphologic remission after completion of induction therapy, consistent with standard AML response criteria. Mutation status for five recurrently altered genes in AML, that is, TP53, NPM1, RUNX1, ASXL1, and FLT3‐ITD were classified as either mutated or wild‐type. Age group was analyzed as a categorical variable using indicator coding in multivariable models. This approach allowed us to assess how genetic alterations and gene expression patterns are associated with treatment response and survival in the same patient population.

### 2.2 Gene Selection and Expression Data Processing

We selected five recurrent AML mutations together with a biologically curated panel of 12 apoptosis and p53‐pathway genes based on established mechanistic relevance to leukemic cell survival and treatment response. This targeted feature selection strategy was chosen to enhance clinical interpretability and minimize high‐dimensional overfitting while enabling mechanistic interpretation of model attribution analysis. We focused on 12 genes with well‐recognized roles in apoptosis and the p53 pathway: BCL2, MCL1, BAX, BCL2L1, BCL2L11, PMAIP1, TP53, CDKN1A, MDM2, GADD45A, CHEK2, and CCNG1. These were selected based on published data linking them to AML biology and treatment response [18, 19, 20, 21, 22, 23, 24, 25, 26, 27, 28]. BCL2 and MCL1 are central anti‐apoptotic regulators, and their overexpression has been associated with reduced sensitivity to venetoclax and chemotherapy [18, 24]. In contrast, BAX, BCL2L1, and BCL2L11 drive pro‐apoptotic mitochondrial signaling, facilitating cell death after cytotoxic stress [20, 25]. PMAIP1 encodes NOXA, which promotes degradation of MCL1 and shifts the balance toward apoptosis [26]. TP53 is a key tumor suppressor that integrates DNA damage signals and determines whether cells undergo repair, arrest, or apoptosis. Its downstream targets that is CDKN1A, MDM2, GADD45A, CHEK2, and CCNG1, carry out these effects through cell cycle regulation, p53 feedback control, DNA repair, checkpoint activation, and growth arrest [22, 27, 28]. Disruption of this pathway, especially through TP53 mutation, is linked to primary treatment resistance and poor outcomes in AML, underscoring the clinical relevance of these genes for our analysis. Likewise, we used normalized RNA‐seq expression data from the BeatAML2 dataset, mapping Ensembl gene identifiers to gene symbols based on the annotation files provided in the dataset. Clinical variables and expression profiles were linked using the database of Genotypes and Phenotypes (dbGaP) sample mapping to ensure patient‐level correspondence between datasets. Gene expression values were obtained from normalized log2 RNA‐sequencing counts provided by the BeatAML processing pipeline. For visualization analyses, expression values were standardized as *z*‐scores according to Equation 1 below, where $x_{i}$ represents the normalized log2 expression value of a given gene for specimen i, μ denotes the cohort mean expression for that gene, and σ represents the corresponding cohort standard deviation.

(1) $$\;z_{i}=\frac{x_{i}-\mu }{\sigma }\;\;$$

### 2.3 Machine Learning Model Development, Evaluation, Sensitivity, and SHAP Analysis

Three machine learning approaches were compared for both CR and 2‐year OS: (1) Logistic regression with L2 regularization, (2) Random Forest (RF) with 500 trees, tuned for maximum depth and balanced class weights, and (3) Extreme Gradient Boosting (XGBoost) optimized for binary classification with log‐loss. The dataset was grouped using a stratified 80/20 training‐test split. Model hyperparameters were optimized using cross‐validated grid search, with final parameters selected based on validation AUC performance. To account for the data imbalance, class‐weighting strategies were applied during model training. Balanced class weights were used for RF and logistic regression models, while the XGBoost algorithm incorporated class imbalance through adjustment of the scale_pos_weight parameter calculated from the training‐set class distribution. In this study, hereafter, we refer to the models incorporating the five mutations and age inputs as baseline models, and to those additionally including the 12 apoptosis‐related gene inputs as enriched models. Model performance was assessed using 5‐fold cross‐validation with stratification by outcome. The primary metric for discrimination was the area under the receiver operating characteristic curve (AUC). Calibration was evaluated using the Hosmer–Lemeshow *χ* ^2^ statistic, which tests agreement between predicted and observed outcomes across risk deciles, and the Brier score, which quantifies overall prediction error as the mean squared difference between predicted probabilities and observed outcomes (lower values indicate better accuracy). Other performance metrics included calibration slope, net reclassification index (NRI), and integrated discrimination improvement (IDI).

Similarly, univariable and multivariable Cox proportional hazards models were performed to evaluate the independent effects of mutations and gene expression on 2‐year OS and CR. Hazard ratios (HR), odds ratios (OR), and 95% confidence intervals (CI) bootstrapped with 1000 iterations were reported. Statistical significance was defined as *p* < 0.005 to minimize false discovery in the context of multiple testing. Robustness of results was evaluated in several ways: (1) repeating models without class weights; (2) restricting predictors to mutations only; (3) sequentially removing single features to assess redundancy; and (4) performing subgroup analyses across the four age groups. For CR and 2‐year OS, NRI and IDI were calculated to quantify reclassification improvement when apoptotic genes were added. To ensure biological interpretability, SHapley Additive exPlanations (SHAP) values were calculated for each model. SHAP feature importance was calculated using the training dataset to obtain stable estimates of how each feature contributed to the model predictions. Feature importance rankings were shown to identify mutations and genes driving predictions. We specifically assessed whether apoptotic regulators contributed consistently alongside canonical mutations such as TP53 and NPM1.

## 3 Results

### 3.1 Cohort Characteristics and Age Distribution

**FIGURE 1:**
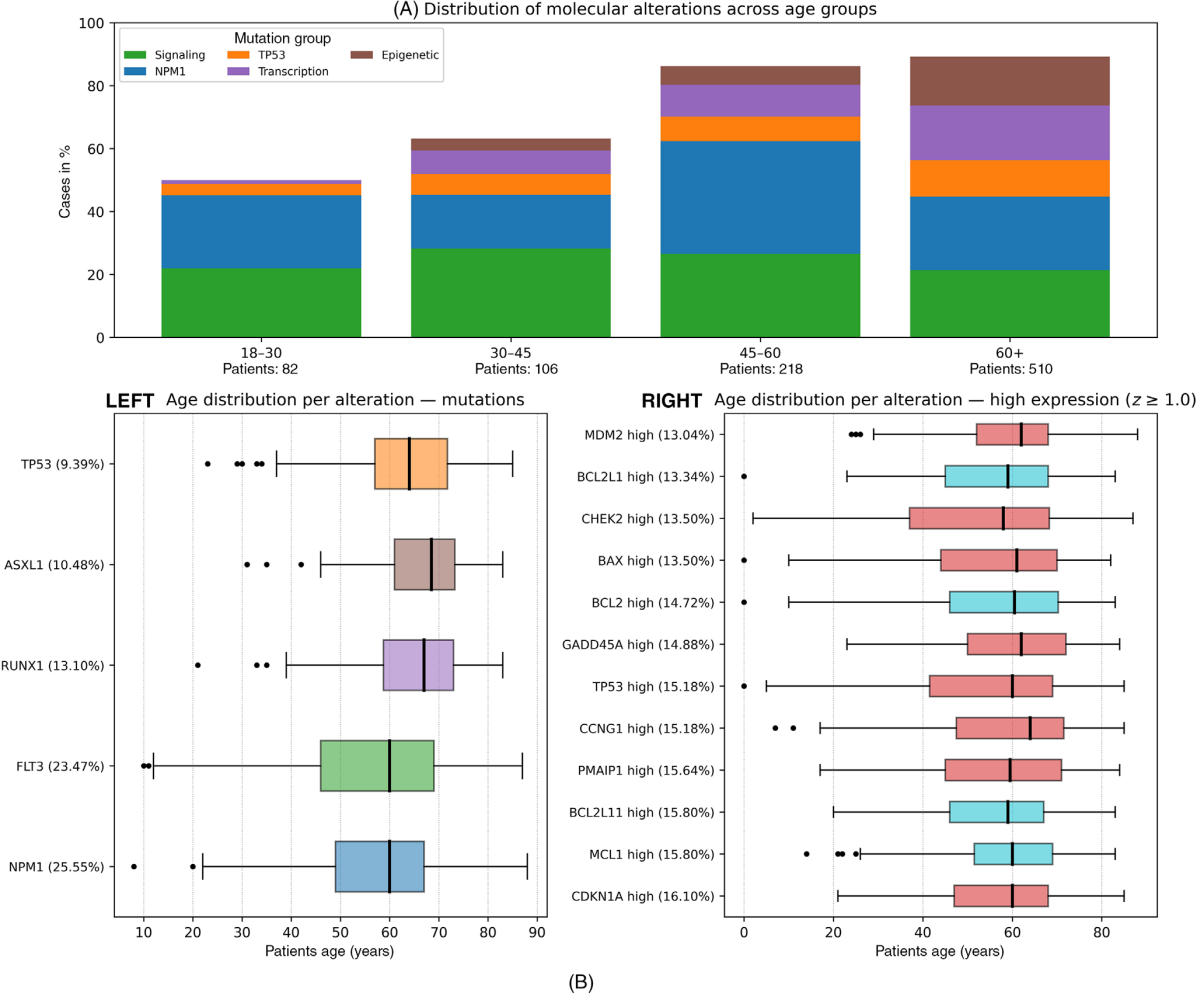
Age‐stratified distribution of recurrent genomic alterations in AML. (A) Stacked bar plots showing the proportion of cases harboring mutations in major functional groups (signaling, NPM1, TP53, transcription‐factor, and epigenetic regulators) across age categories 18–30, 30–45, 45–60, and 60+ years. (B) LEFT: Boxplots depicting the distribution of patient age for each recurrent mutation; RIGHT: Boxplots depicting the distribution of patient age among cases with high expression (*z* ≥ 1.0) of selected apoptosis‐related genes.

We analyzed a total of 916 specimens of patients with mutation data and 852 specimens with paired RNA‐seq expressions and clinical outcome data. The median age at diagnosis was 58 years (interquartile range [IQR]: = 46–67), and more than half of the cohort that is 56% were aged 60 years or older. This distribution reflects the natural incidence of AML, where older adults account for the majority of diagnoses and carry the poorest outcomes. The rate of CR following induction was 62.1%, while only 19% of patients remained alive at 2 years, highlighting the dismal long‐term prognosis that persists despite current therapeutic options. Molecular analysis revealed clear age‐related patterns. Older patients carried higher frequencies of TP53 and epigenetic mutations, while younger patients more often harbored signaling mutations such as FLT3 and favorable mutations such as NPM1. At the transcriptomic level, apoptosis‐related genes also demonstrated differences across age, raising the hypothesis that apoptotic genes may act as an independent determinant of outcomes beyond classical mutation status. Figure 1 illustrates these distributions, demonstrating how the biology of AML shifts with age in ways that may alter prognosis and treatment sensitivity.

### 3.2 Combined Mutational and Gene Expression Predictors of Remission and Survival

Across the entire cohort, TP53 mutations emerged as the single strongest predictor of adverse outcomes. In multivariable models, TP53 was associated with a 3‐fold increased risk of death (HR 3.07, 95% confidence interval [CI]: 2.29–4.12, *p* < 0.005). The adverse impact was observed in both younger (< 60 years) and older adults (> 60 years), though its magnitude differed. In patients younger than 60 years, TP53‐mutated AML was associated with significantly poor survival (HR 11.49, 95% CI: 5.58–23.64, *p* < 0.005), while in older adults the risk remained substantial but less extreme (HR 2.53, 95% CI: 1.79–3.56, *p* < 0.005). In absolute terms, median OS for TP53‐mutated older adults was only 4.8 months compared with 18.2 months in their wild‐type counterparts. These results underscore why TP53‐mutated AML continues to be regarded as a distinct clinical entity, and why age‐specific context is essential when interpreting risk. Similarly, the other four recurrent mutations contributed to prognosis as well. ASXL1 mutations carried a 1.5‐fold increased risk of death (HR 1.53, 95% CI: 1.18–1.99, *p* < 0.005), and FLT3 mutations were also associated with inferior survival (HR 1.39, 95% CI: 1.13–1.71, *p* < 0.005). For remission outcomes, NPM1 mutations conferred a more than 2‐fold increased likelihood of achieving CR (OR 2.47, 95% CI: 1.16–5.30, *p* = 0.02), although this did not translate into improved long‐term survival, consistent with prior reports of context‐dependent benefits [29, 30, 31].

**FIGURE 2:**
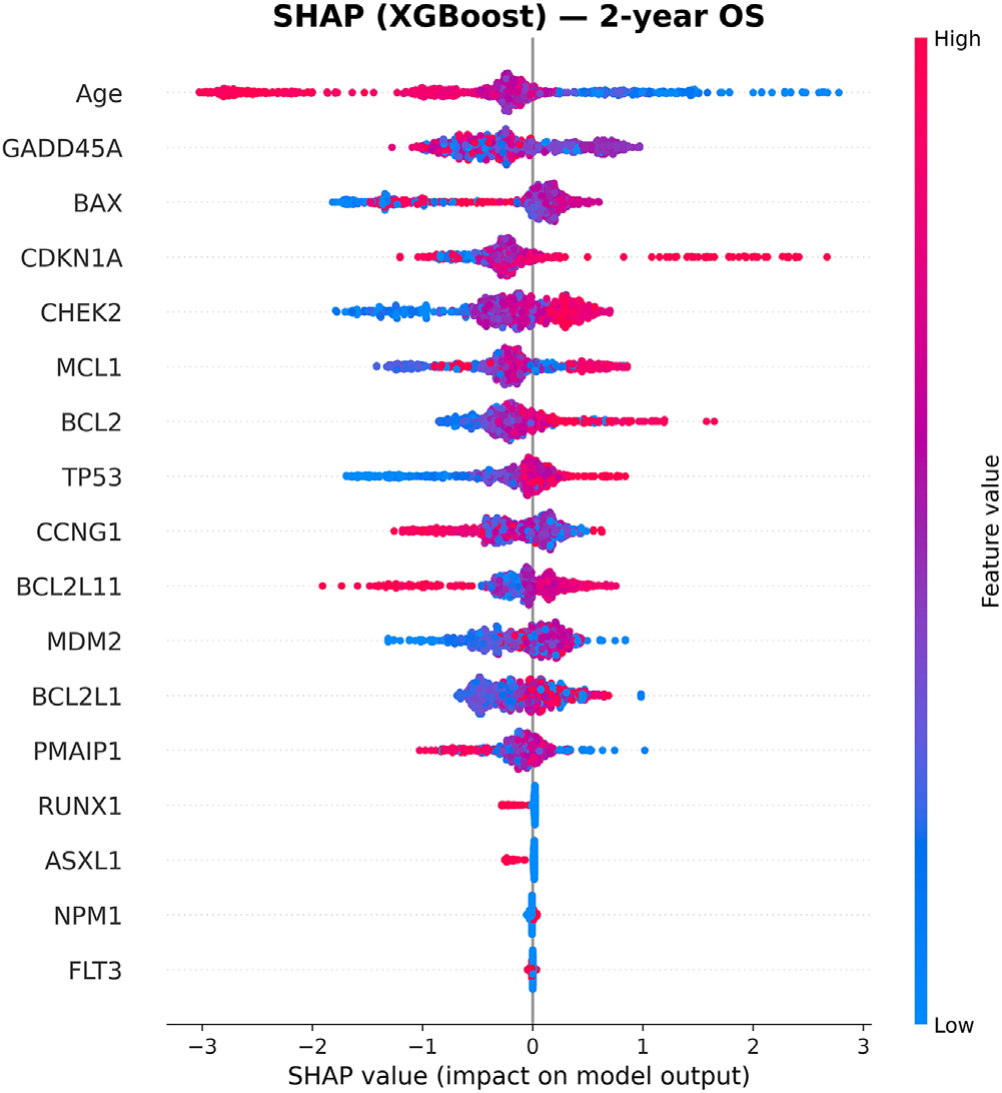
SHAP summary plot for the XGBoost model predicting 2‐year OS. Age was the dominant predictor. CHEK2 and CDKN1A expression contributed strongly to OS prediction, while recurrent mutations had comparatively smaller effects.

**FIGURE 3:**
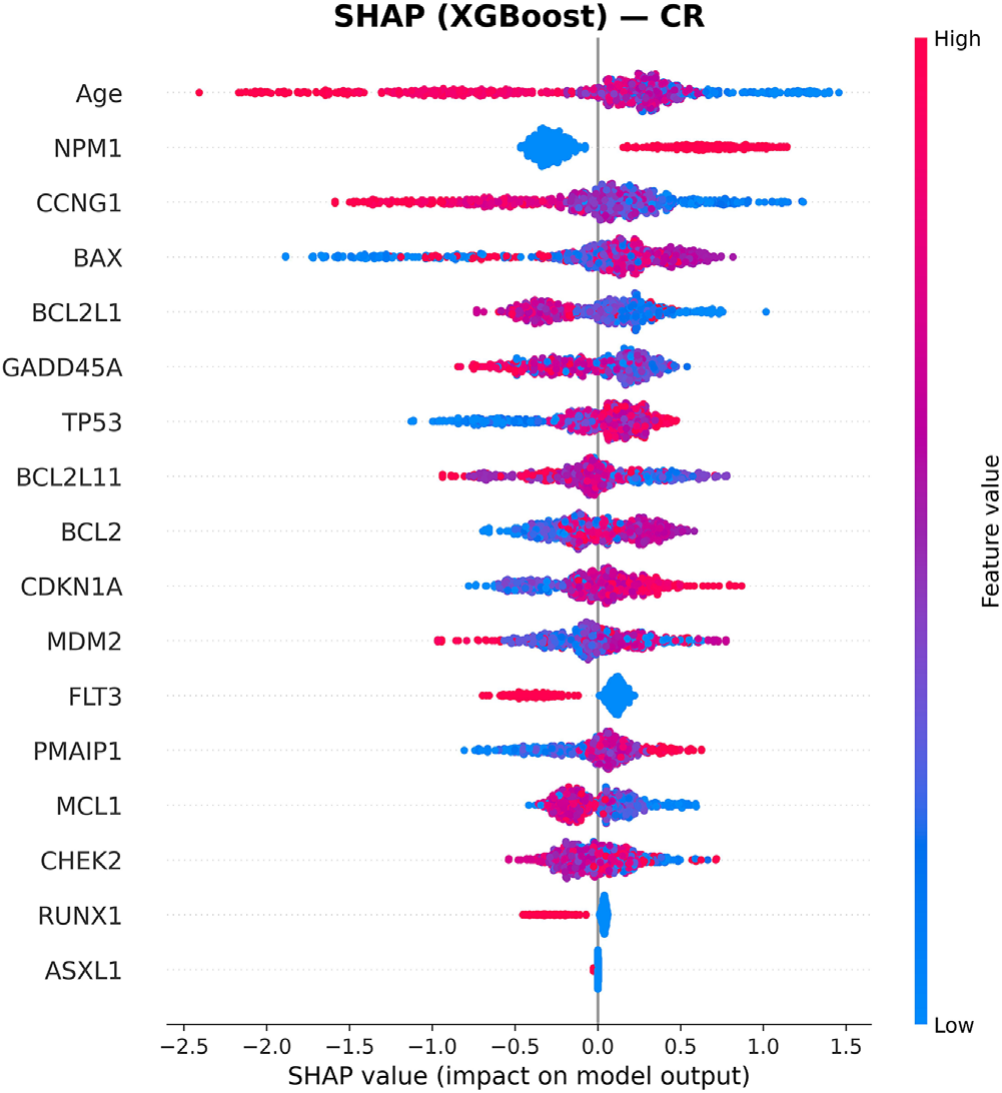
SHAP summary plot for the XGBoost model predicting CR. Age and NPM1 mutation status were the strongest contributors. CCNG1 and BAX expressions further influenced CR predictions.

**FIGURE 4:**
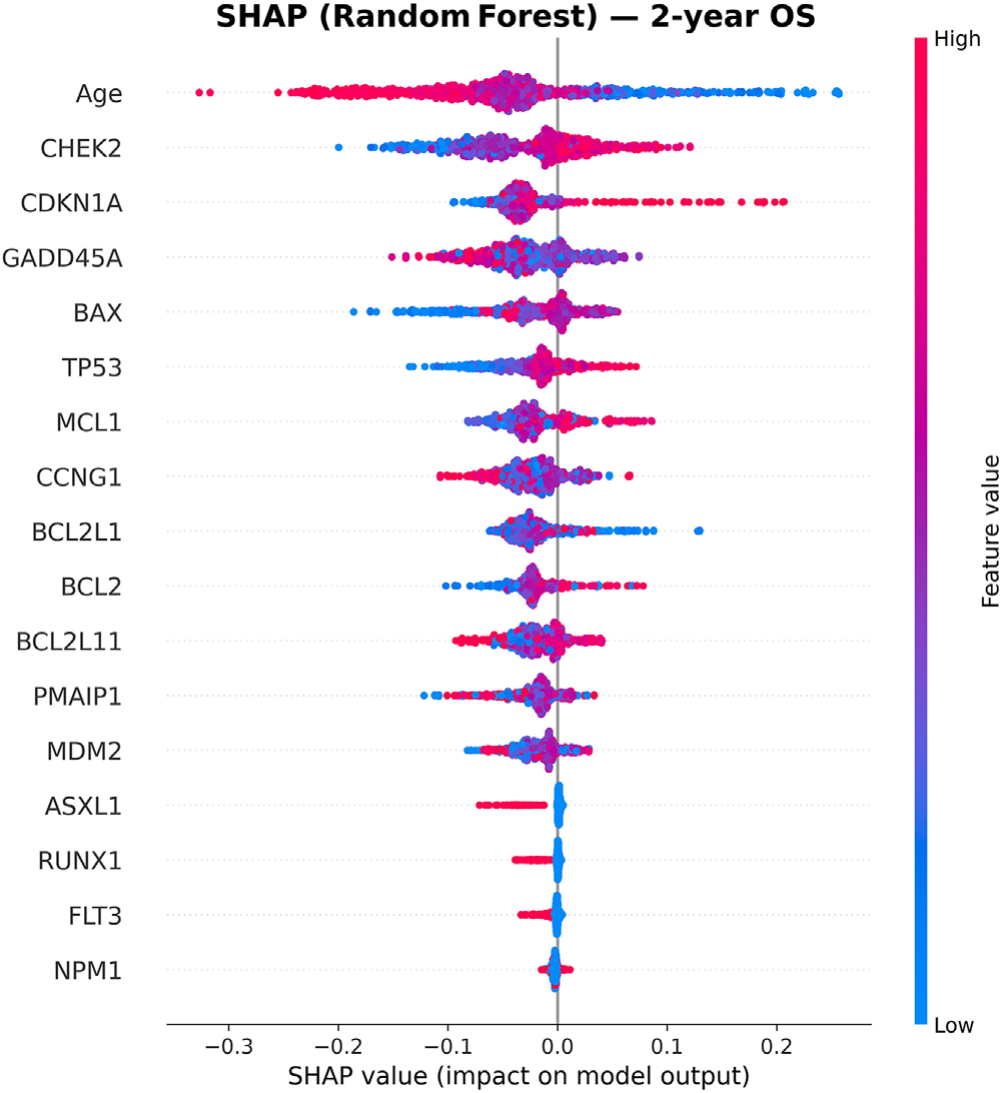
SHAP summary plot for the Random Forest model predicting 2‐year OS. Age remained the leading feature, with CHEK2 and CDKN1A expression providing additional prognostic value beyond mutation status.

**FIGURE 5:**
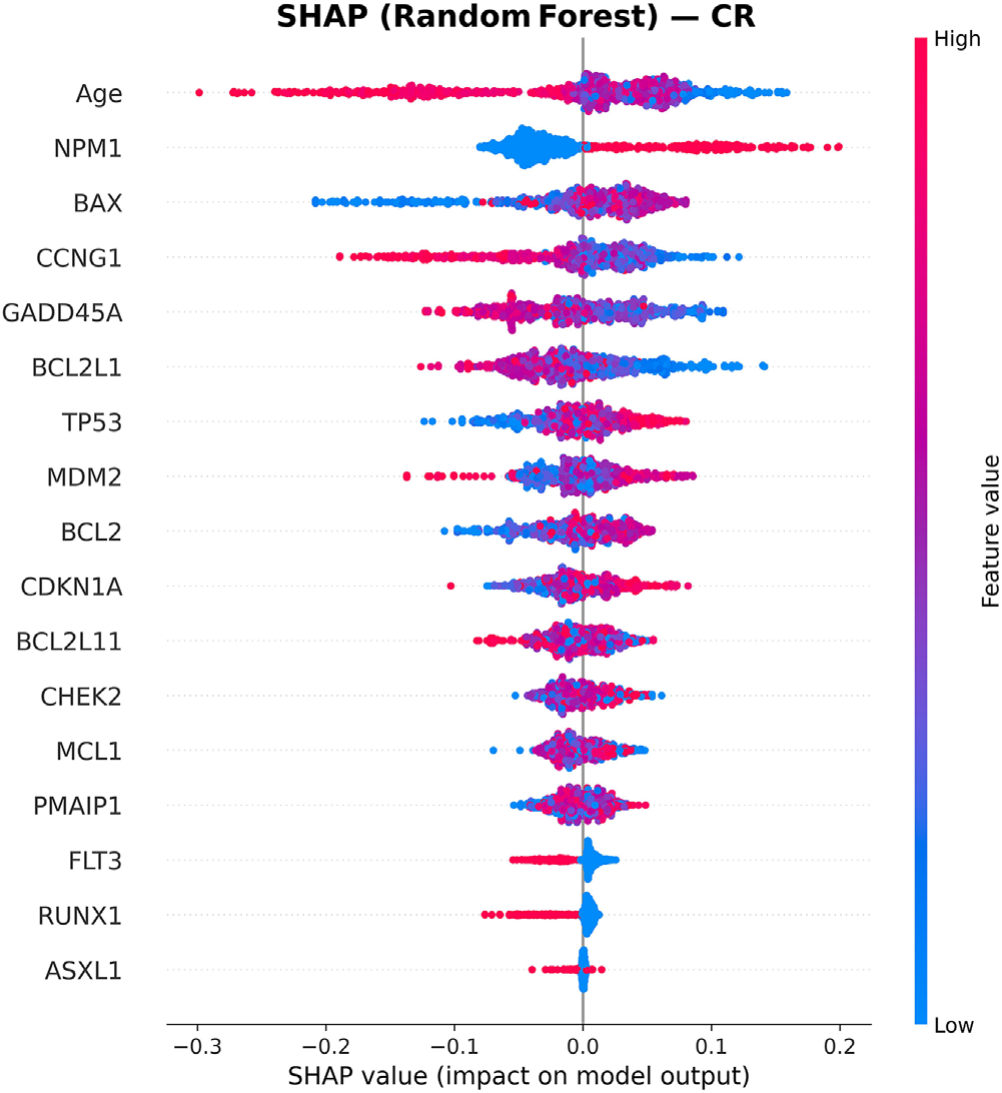
SHAP summary plot for the Random Forest model predicting CR. Age and NPM1 mutation status were dominant predictors, with apoptosis‐related gene expression refining remission prediction.

To move beyond mutation‐only predictors, we incorporated expression of 12 apoptosis and p53 pathway genes (BAX, CCNG1, GADD45A, CDKN1A, MDM2, TP53, PMAIP1, MCL1, BCL2L11, BCL2L1, BCL2, and CHEK2). This panel added complementary information that significantly improved model discrimination. Within Cox models, higher MCL1 expression was independently protective (HR 0.81, 95% CI: 0.70–0.94, *p* < 0.005), as was higher BCL2 expression (HR 0.88, 95% CI: 0.78–0.99, *p* = 0.034). Most importantly, these effects were consistent across age groups, indicating that apoptotic genes carried stable prognostic value even in older adults, who represented the majority of our cohort. This has direct clinical relevance, as risk prediction in older AML patients, particularly 60+ age group, is often limited when relying on mutations alone [32]. Figures 2, 3, 4, 5 illustrate these relationships, where age was the predominant predictor for both 2‐year OS and CR across XGBoost and RF models. For a 2‐year OS, CHEK2 and CDKN1A expression contributed strongly, particularly to the RF model, while TP53 mutation had a comparatively modest effect, as shown in Figure 4. For CR, age and NPM1 mutation status were dominant predictors, with BAX and CCNG1 expressions also showing strong contributions across both models. In a sensitivity analysis modeling age as a continuous variable, age remained independently associated with OS. Each 1‐year increase in age corresponded to an approximately 2.4% increase in the hazard of death (HR: 1.024, 95% CI: 1.024–1.030, *p* < 0.001), consistent with prior AML prognostic modeling studies that treat age as a continuous covariate. Similarly, for CR, the NPM1 mutation was the strongest predictor, with CCNG1 expression ranking highest among the expression features, alongside BAX, MCL1, and BCL2 family members. Overall, these results demonstrate that in addition to canonical mutations, gene expression features such as CHEK2 and CCNG1 add meaningful prognostic information, reinforcing the value of integrating gene expression data into AML risk models.

**FIGURE 6:**
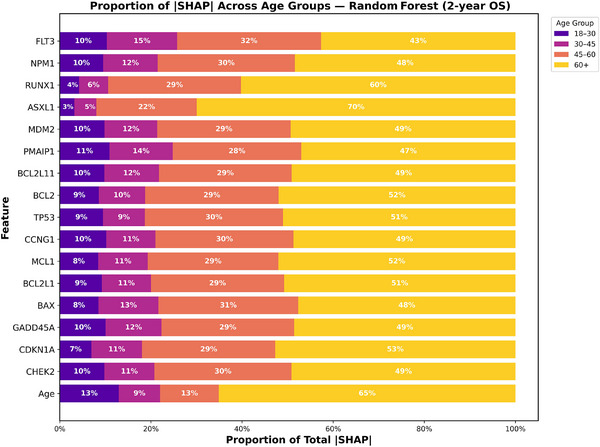
XGBoost Age‐stratified SHAP proportion plot for 2‐year OS. Stacked bar plots show the proportion of patients within each age group (18–30, 30–45, 45–60, and 60+) for the top‐ranked predictors.

**FIGURE 7:**
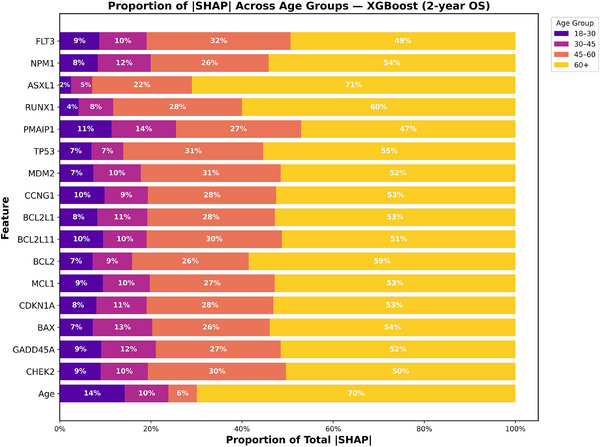
XGBoost age‐stratified SHAP proportion plot for CR.

**FIGURE 8:**
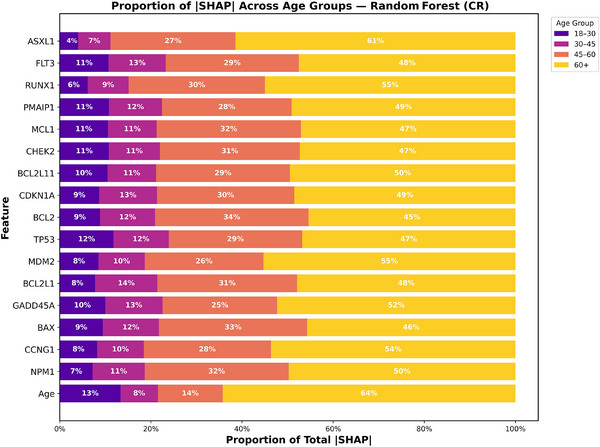
Random Forest age‐stratified SHAP proportion plot for 2‐year OS.

**FIGURE 9:**
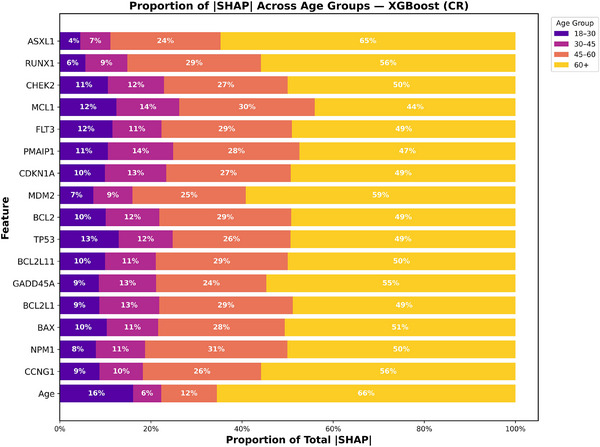
Random Forest age‐stratified SHAP proportion plot for CR.

To better understand how prognostic contributions varied across age groups, we analyzed SHAP value proportions stratified by age for both 2‐year OS and CR for XGBoost and RF models as shown in Figures 6, 7, 8, 9. For both the XGBoost and RF models in survival and remission prediction, age itself remained the single largest contributor. Similarly, for 2‐year OS, RF models showed that CHEK2 expression accounted for nearly 30% of the predictive contribution in patients aged 45–60 and increased further to 49% in 60+ age group, whereas younger groups contributed less than 15%. XGBoost models showed a similar pattern, with CHEK2 and TP53 retaining strong influence but with the greatest proportional weight carried by older adults. This enrichment of SHAP weight in the 60+ age group reflects both the overrepresentation of older patients in our cohort (nearly 60% of the total population) and the biology of AML in older age, where TP53 dysfunction, apoptotic dysregulation, and complex genetic backgrounds are more frequent, making these features highly discriminative for survival. For CR prediction, NPM1 mutation and CCNG1 expression consistently dominated across both models. In RF model, CCNG1 explained about 28% of predictive weight in the 45–60 age group, rising to 54% in 60+ age group, again indicating stronger predictive utility of apoptotic signaling in older AML patients. XGBoost yielded similar distributions, with BAX and MCL1 additionally ranking high, underscoring the influence of apoptosis regulators on remission outcomes. Overall, these findings show that while recurrent mutations remain essential, expression‐based markers such as CHEK2 and CCNG1 provide added discriminatory value precisely in older adults, where traditional mutation‐only risk models often lose out to some degree.

### 3.3 Added Value of the 12‐Gene Apoptosis Panel on Discrimination, Calibration, and Clinical Utility

**FIGURE 10:**
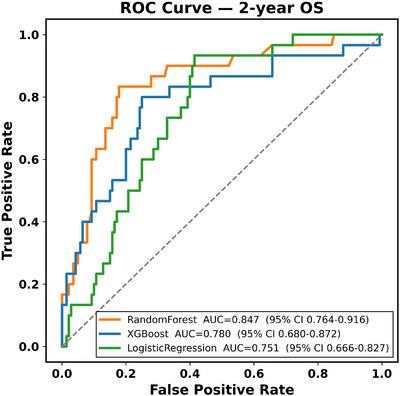
ROC curves for prediction of 2‐year OS.

**FIGURE 11:**
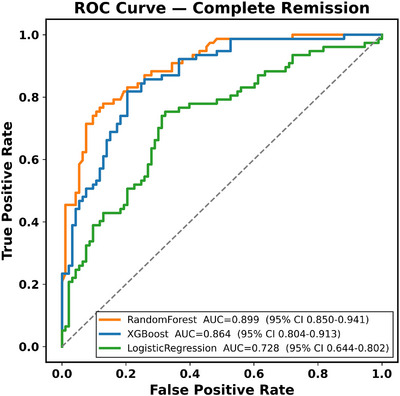
ROC curves for prediction of CR.

We evaluated whether incorporating 12 apoptotic gene expressions improved model performance beyond age and recurrent mutations. For 2‐year OS, baseline models with age and mutation inputs achieved AUCs of 0.765 for XGBoost (95% CI: 0.665–0.850), 0.703 for RF (95% CI: 0.591–0.807), and 0.697 for logistic regression (95% CI: 0.606–0.778). After adding the 12‐genes, performance increased to 0.772 for XGBoost (95% CI: 0.675–0.865), 0.843 for RF (95% CI: 0.776–0.911), and 0.721 for logistic regression (95% CI: 0.634–0.802). The largest gain was observed for RF, which improved by +0.14 AUC, highlighting that tree‐based machine learning approach was able to capture added prognostic information from gene expression to the greatest extent. For CR, enrichment models produced even more pronounced gains. Baseline AUCs were 0.770 for XGBoost (95% CI: 0.698–0.839), 0.811 for RF (95% CI: 0.744–0.878), and 0.731 for logistic regression (95% CI: 0.652–0.804). With the gene panel, discrimination improved to 0.851 for XGBoost (95% CI: 0.789–0.904) and 0.861 for RF (95% CI: 0.804–0.909), representing increases of +0.08 and +0.05 AUC, respectively. Logistic regression did not benefit, with performance falling slightly to 0.696 (95% CI: 0.614–0.774). Figures 10, 11 show the ROC curves for 2‐year OS and CR prediction, where RF consistently achieved the highest discrimination compared with XGBoost and Logistic Regression. In a sensitivity analysis evaluating 1‐year OS, model discrimination was higher, with RF achieving an AUC of 0.915 and XGBoost attaining 0.886. This finding is clinically expected in AML, where mortality events are heavily concentrated within the first year after diagnosis and are therefore more closely linked to baseline disease biology and early treatment response. By contrast, 2‐year OS is influenced by additional post‐remission factors, including consolidation strategies, transplantation, salvage therapies, and supportive care, which introduce greater clinical heterogeneity and make longer time horizon predictions inherently more challenging. However, despite this increased complexity, the addition of the 12‐gene apoptosis and p53‐pathway panel produced the largest relative improvement in predictive performance for the 2‐year OS, precisely where prognostication is most difficult. These observations indicate that early survival reflects predominantly baseline molecular risk, whereas longer‐term outcomes incorporate additional therapeutic and clinical determinants, nevertheless, the integrated mutation and expression models retained strong discrimination across both 1‐year and 2‐year OS, supporting their clinical relevance for risk stratification in AML. Calibration analyses supported the improvements with both XGBoost (Brier = 0.158, HL *χ* ^2^ = 5.72, *p* = 0.679) and RF (Brier = 0.153, HL *χ* ^2^ = 10.63, *p* = 0.223) demonstrating decent agreement between predicted and observed outcomes, while logistic regression remained less well calibrated (Brier = 0.224, HL *χ* ^2^ = 10.80, *p* = 0.213). Reclassification analysis highlighted differences between models. Adding the gene panel improved patient‐level classification for XGBoost (NRI = 0.511, 95% CI: 0.218–0.792; IDI = 0.105, 95% CI: 0.036–0.171). By contrast, RF and logistic regression did not improve reclassification, reflecting that their discrimination gains were captured by better calibration rather than shifting patients between categories. Subgroup analyses confirmed that the added value of gene expression was not restricted to any single age group. For RF, the enriched model achieved AUCs of 0.889 in patients 18–30 years, 0.657 in those 30–45 years, 0.960 in 45–60 years, and 0.846 in patients ≥ 60 years. The test for risk‐by‐age interaction was not significant (*χ* ^2^ = 5.29, *p* = 0.152), indicating that improvements were consistent across age strata despite small sample sizes in younger cohorts. Finally, sensitivity analyses demonstrated the robustness of these findings. Removing class weighting had minimal effect (AUC 0.858), while restricting RF to mutations only reduced AUC to 0.689. Dropping any single mutation feature did not meaningfully alter discrimination (AUCs remained 0.853–0.863), underscoring that predictive gains were driven by the integrated panel rather than dependence on one single driver.

## 4 Conclusion

We studied five common AML mutations (TP53, NPM1, FLT3, RUNX1, and ASXL1) together with 12 genes (BAX, CCNG1, GADD45A, CDKN1A, MDM2, TP53, PMAIP1, MCL1, BCL2L11, BCL2L1, BCL2, and CHEK2) involved in apoptosis and the p53 pathway. The goal was to see how these features, alone and together, predict both CR and 2‐year OS across four age groups (18–30, 30–45, 45–60, and 60+). The mutation findings were clear. TP53 was the strongest adverse marker, with patients carrying this mutation having more than a three times higher risk of death. The effect was most severe in younger patients aged 18–30 and 30–45, where HR were over 10, but the mutation also had a strong effect in patients aged 60 and older, who had a median survival of under 5 months. ASXL1 and FLT3 mutations were also linked with worse outcomes, especially in patients aged 45–60. NPM1 mutation improved chances of remission in every age group, most strongly in patients 30–45, but this did not lead to longer survival. RUNX1 showed weaker effects in all three machine learning models (both baseline and enriched models), especially in patients aged 60 and older, but it remains important clinically because of its link to secondary AML and complex karyotypes. Similarly, FLT3 had little influence on the machine learning models, yet its clinical relevance should not be overlooked. An important point we would like to make here is that although the machine learning downplayed some mutations in this study, their clinical value remains and should be paid high importance while building predictive models.

Similarly, on adding gene expression, we attained newer insights, for 2‐year OS, CHEK2 was one of the strongest features in both enriched RF and XGBoost models, accounting for about 30% of importance in patients aged 45–60 and nearly 50% in patients aged 60 and older in RF. This fits its role in DNA repair and apoptosis, processes often disrupted in older AML. CDKN1A, GADD45A, and BAX also contributed, highlighting the role of apoptosis and cell‐cycle control in survival. For CR, NPM1 and age remained the top predictors, but CCNG1 expression was especially important in patients 45–60 and 60+, with BAX and TP53 also adding weight. In almost every plot, the ≥ 60 age group carried the greatest share of predictive weight across both mutations and genes. This reflects both the larger number of older patients and the heavier disease burden in this group. It also highlights why enriched models are most useful in older AML, where treatment decisions are hardest. Taken together, five features stood out across all analyses: Age, TP53, NPM1, CHEK2, and CCNG1. These represent both well‐known clinical markers and new biological signals, and together they improved prediction and calibration of the models.

There are some limitations of our study worth discussing. In the BeatAML2 dataset, since the treatment strategies varied across the cohort, survival outcomes may also reflect therapeutic heterogeneity. Likewise, while splitting patients into four age groups, we were left with some smaller subgroups, which makes very detailed comparisons harder. Even so, this approach allowed us to highlight how risk factors vary across the different age groups, especially in older patients who formed the largest part of the cohort. We also focused on only the five established mutations and a 12‐gene panel rather than on every AML expression. Our reasoning for this was that limiting the number of features in a machine learning model would make it easier for us to interpret and demonstrate more clearly how certain combinations of mutations and gene expressions would improve prediction. We also did not include treatment details such as transplant or targeted therapy, which reflects the variability of real‐world care. However, we believe that future studies, especially those done in clinical trial settings, can build on our results under more controlled conditions.

## Author Contributions

**Mobina Shrestha**: conceptualization, investigation, data analysis and visualization, data verification, methodology, writing – original draft. **Salina Dahal**: investigation, data analysis and visualization, writing – review and editing. **Asis Shrestha**: data verification, methodology. **Patricia McNally**: writing – review and editing, supervision. **Niklas Mackler**: writing – review and editing. All authors read and approved the final manuscript.

## Funding

The authors have nothing to report.

## Ethics Statement

Data used in this study were obtained from the BeatAML2 cohort, which is a public and anonymous dataset, so Institutional Review Board approval was not required.

## Consent

As this study is retrospective in nature and involves the analysis of existing BeatAML2 data, the requirement for informed consent was waived by the ethics committee. However, informed consent was obtained from all individual participants included in the BeatAML2 study.

## Conflicts of Interest

The authors declare no conflicts of interest.

## Data Availability

The data that support the findings of this study are available on request from the corresponding author.
